# Association of handgrip strength with chronic diseases and multimorbidity: A cross-sectional study

**DOI:** 10.1007/s11357-012-9385-y

**Published:** 2012-02-08

**Authors:** Ching-Lung Cheung, Uyen-Sa D. T. Nguyen, Eleanor Au, Kathryn C. B. Tan, Annie W. C. Kung

**Affiliations:** 1 Department of Medicine, University of Hong Kong, Pokfulam, Hong Kong China; 2 Department of Psychiatry, University of Hong Kong, Pokfulam, Hong Kong China; 3 Department of Medicine, Clinical Epidemiology Research and Training Unit, Boston University School of Medicine, Boston, MA USA; 4 Intensive Care Unit, St. George’s Hospital, London, UK

**Keywords:** Handgrip strength, Multimorbidity, Chronic disease, Association

## Abstract

The prevalence of chronic diseases has risen along with increased longevity. Co-occurrence of two or more chronic diseases in an individual (multimorbidity) is prevalent and poses a huge burden to individuals and the society. However, determinants of multimorbidity are largely unknown. Handgrip strength is a general indicator of muscle strength and linked with premature mortality. However, its role in multimorbidity has never been evaluated. To investigate the relationships between handgrip strength and multiple chronic diseases and multimorbidity, and to assess the usefulness of age and handgrip as a marker of chronic diseases and multimorbidity in a community dwelling sample of men and women, we analyzed a cross-sectional cohort with 1,145 subjects (748 men and 397 women) aged 50 years and older living in Hong Kong. Low handgrip strength was significantly associated with increased odds of having five and three chronic diseases in men and women, respectively, after controlling for age, body mass index, history of smoking, educational level, marital level and comorbidity. Multivariable-adjusted handgrip strength was significantly decreased with the number of chronic diseases in men (trend, *P =* 0.001), but the trend in women was marginal (trend, *P* = 0.06). Conversely, multivariable-adjusted age was significantly increased with the number of chronic diseases in women (trend, *P* = 0.033), but not in men (trend, *P* = 0.118). In conclusion, handgrip strength is associated with multiple chronic diseases and multimorbidity in men and women after adjustment of confounding factors. It shows a linear trend of association with the number of chronic diseases in men, but not in women. Since handgrip strength is a biomarker of multiple physiological systems, its augmentation may be a feasible strategy to improve general health and decrease likelihood of having multiple chronic diseases and hence, premature mortality.

**Electronic supplementary material:**

The online version of this article (doi:10.1007/s11357-012-9385-y) contains supplementary material, which is available to authorized users.

## Introduction

The prevalence of chronic diseases has risen along with longevity (Knottnerus et al. 1992), and the prevalence is expected to rise further owing to an aging population. In addition, people usually suffer from more than one chronic disease. There are two terms describing the disease co-occurrence: comorbidity and multimorbidity. Comorbidity refers to having a number of other diseases beyond the index disease under study. In contrast, multimorbidity is defined as any co-occurrence of two or more chronic diseases within the same person (van den Akker et al. 1996).

Multimorbidity is highly prevalent and poses a huge burden on individuals and society. In a general population of 13,584 Dutch subjects, the prevalence of multimorbidity in subjects aged 65–74 years was 30% and in those aged 75 years or above was 55% (Schram et al. 2008). Previous studies have shown that multimorbidity increases the mortality risk (Marengoni et al. 2011; Newman et al. 2008), causes a decline of physical and mental functioning (Marengoni et al. 2009, 2011), and adversely influences quality of life (Fortin et al. 2006; Marengoni et al. 2011; Tooth et al. 2008). Additionally, it is also associated with needing help with activities of daily living, longer hospitalization, and higher overall health service utilization (Marengoni et al. 2011; Tooth et al. 2008).

Although multimorbidity poses a major problem to individuals, health care providers and society, research of multimorbidity is still in its infancy (Fortin et al. 2005, 2007). A few factors have been shown to be associated with multimorbidity. For example, age is a well-known determinant of multimorbidity, as the number of co-existing diseases in a person rises with age (Marengoni et al. 2008; Schellevis et al. 1993; van den Akker et al. 1998). Female gender, lower socioeconomic status, lower education, smoking, high waist circumference, and separated or divorced or widowed in marital status were also associated with increased risk of multimorbidity (Marengoni et al. 2008; Taylor et al. 2010). However, biomechanical determinants of multimorbidity are still poorly understood. Despite recent research interests in handgrip strength, the association between handgrip strength and multimorbidity has, to our knowledge, never been evaluated.

Handgrip strength is a general indicator of muscle strength, and low handgrip strength has been linked with premature mortality in middle-aged and elderly subjects (Metter et al. 2002; Takata et al. 2007). In addition, handgrip strength may serve as a biomarker of other systems, such as the endocrine system. A randomized controlled trial reported that sex steroid plus growth hormone intervention significantly increased muscle strength in men, but not in women (Blackman et al. 2002), and this observation was further supported by a meta-analysis (Ottenbacher et al. 2006). These observations suggested that handgrip strength may be controlled by multiple physiological systems. Earlier studies suggested that handgrip strength is associated with a number of chronic diseases (Fried et al. 2001; Karkkainen et al. 2008; Oken et al. 2008; Sayer et al. 2007; Syddall et al. 2003). However, many of these studies were based on a single disease paradigm and did not account for comorbidity or multimorbidity. Therefore, the first aim of the current study was to evaluate the relationship between handgrip strength and each individual chronic disease with adjustment for comorbidity. The second aim was to investigate whether handgrip strength was associated with multimorbidity, and if it was better than age as a marker of multimorbidity.

## Material and methods

This handgrip strength study formed part of the Hong Kong Osteoporosis Study which was initiated in 1995. The population cohort participants were community-dwelling Southern Chinese men and women recruited from public road shows and health fairs held in various districts of Hong Kong. From 1998 to 2009, a total of 9,353 southern Chinese men and women were recruited. Handgrip strength data were first collected at the end of 2002. A total of 4,861 subjects had handgrip strength data. Subjects under the age of 50 (*n* = 2,025) were excluded, as were subjects who refused to answer the section on the questionnaire regarding medical history (missing total sections of the medical history) (*n* = 1,574). In the remaining 1,262 subjects, 58 men and 59 women with at least one missing data (on any chronic disease) were also excluded. Missing data were due in large part to subjects being uncertain whether they suffered from particular chronic diseases. Data from 748 men and 397 women were analyzed in the present study. Study recruitment and eligibility flow chart is provided in Fig. S1. All participants gave informed consent and the study was conducted according to the Declaration of Helsinki. The study protocol was approved by the Institutional Review Board of the University of Hong Kong and the Hospital Authority Hong Kong West Cluster Hospitals.

### Collection of clinical data and selection of chronic diseases

In brief, baseline demographic data on anthropometric measurements, socioeconomic status, education level, and medical and reproductive history were obtained using a structured questionnaire administered by a research nurse or assistant. The medical record was confirmed by using the computerized patient record system of the Hong Kong Hospital Authority, which manages outpatient clinics and hospitals attended by the majority (94%) of Hong Kong population. Lifestyle and dietary habits, including smoking, alcohol consumption, and physical activity, were also recorded. Details of this have been described previously (Cheung et al. 2006; Tang et al. 2009; Tsang et al. 2009).

In the current study, we selected 18 chronic diseases that were present in our database and also prevalent (defined by ≥1%) in our study population. Five chronic diseases (parkinsonism, malabsorption, hyperparathyroidism, dementia, and rheumatoid arthritis) were excluded due to the prevalence of <1%. Stages of chronic kidney disease (CKD) were determined using the estimated glomerular filtration rate (eGFR) and the following equation (Ma et al. 2006):

$$ {\text{eGFR}}\left( {{\text{ml}}/{ \min }\;{\text{per}}\;{1}.{73}\;{{\text{m}}^{{2}}}} \right) = {186} \times {\text{plasma}}\;{\text{creatinine}}{\left( {{\text{mg}}/{\text{dl}}} \right)^{{ - {1}.{154}}}} \times {\text{ag}}{{\text{e}}^{{ - 0.{2}0{3}}}} \times 0.{742 }\left( {{\text{if}}\;{\text{female}}} \right) \times {1}.{233}\left( {\text{if Chinese}} \right) $$

Subjects with eGFR <60 were classified as having CKD stage 3 or above. All other conditions were self-reported by answering survey questions such as “Do you have or have you had…?”

The 18 chronic diseases included: (1) anaemia, 2) anxiety, (3) cataract, (4) cerebral vascular accident (stroke), (5) CKD stage 3 or above, (6) chronic obstructive airways disease, (7) depression, (8) diabetes, (9) history of fall in the past 12 months, (10) hepatitis B, (11) hyperlipidaemia, (12) hypertension, (13) hyperthyroidism, (14) ischemic heart diseases, (15) kyphosis, (16) malignancy within 5 years, (17) osteoarthritis knee, and (18) peptic ulcer. These 18 chronic diseases were based on the 12 categories of the World Health Organization’s International Classification of Diseases (10th Revision, version for 2007, http://apps.who.int/classifications/apps/icd/icd10online/) (Table S1).

### Handgrip strength and covariate assessment

Baseline handgrip strength (in kg) was measured using a dynamometer (Smedley Hand Dynamometer, Stoelting Co, Wood Dale, IL). The test was administered by a trained nurse, and the mean score of three measures in the dominant hand was used in the analysis (Cheung et al. 2011).

Since the aim of our study was to investigate the relationship of handgrip strength and chronic diseases and multimorbidity in an aged population, transformation of the handgrip strength measurements to *T*-scores enabled us to assess handgrip strength with increasing age. We computed a score for standardized handgrip strength for each individual using the formula:

$$ {\text{Standardized}}\;{\text{handgrip}}\;{\text{strength}}\;T\;{\text{score}} = \left( {{\text{handgrip}}\;{\text{strength}}\;{\text{value}} - {\text{reference}}\;{\text{mean}}} \right)/{\text{reference}}\;{\text{standard}}\;{\text{deviation}}\left( {\text{SD}} \right). $$

The age group (30–39 years) with highest mean handgrip strength served as the reference group for the other age groups (Cheung et al. 2011).

Age was obtained in the questionnaire. Height was measured without shoes to the nearest 0.1 cm using a wall-mounted stadiometer. Weight was measured to the nearest 0.1 kg using an electrical scale, with the subject wearing light indoor clothing.

### Statistical analysis

We calculated odds ratios (OR) and 95% confidence interval (CI) for each dichotomized chronic disease using logistic regression. Univariable and multivariable models were performed. In the simple multivariable model (model 2), age, body mass index (BMI), history of smoking, educational level and marital status were adjusted. To eliminate the potential confounding effects of comorbidity, we adjusted for all other chronic diseases in the full model (model 3). We also used analysis of covariance (ANCOVA) to compare least-squares adjusted handgrip strength and chronological age among subjects with increasing number of co-existing chronic diseases and to test for a linear trend. The ANCOVA model included age, handgrip strength, BMI, history of smoking, educational level and marital status. A value of *p* ≤ 0.05 was considered statistically significant. All statistical analyses were performed using SPSS V16.0.2 software.

## Results

On average, men were significantly younger and had higher BMI than women in the study. The mean (SD) handgrip strength and its *T* score were 30.7 (7.9) and −1.22 (0.93) in men, respectively. The corresponding mean (SD) handgrip strength and its *T*-score in women were 16.8 (6.4) and −1.45 (1.25), respectively. Among the 18 chronic diseases, ischemic heart disease was significantly more prevalent in men, while anaemia, cataract, history of fall in the past 12 months and hyperthyroidism were significantly more prevalent in women (Table 1). The prevalence of each chronic disease in studied men and women is shown in Table 1.

**Table 1:** Demographic characteristics and prevalence of each disease in all subjects

	Male		Female		*P* value^a^
	Mean	SD	Mean	SD	
*n*	748		397		
Age (years)	70.7	7.6	72	10.7	0.036
BMI	23.2	3.3	22.1	4.2	<0.001
Handgrip strength (kg)	30.7	7.9	16.8	6.4	<0.001
Handgrip strength *T* score	−1.2	0.9	−1.5	1.3	<0.001
	*n*	%	*n*	%	*P* value^b^
Educational level					<0.001
No	60	8	187	47.1	
Primary	174	23.3	112	28.2	
Secondary	324	43.3	77	19.4	
College or university	190	25.4	21	5.3	
Single	36	4.8	57	14.4	<0.001
Divorced	58	7.8	143	36	<0.001
History of smoker	288	38.5	28	7.1	<0.001
Anaemia	16	2.1	22	5.5	0.003
Anxiety	12	1.6	5	1.3	0.800
Cataract	61	8.2	123	31.0	<0.001
Cerebral vascular accident (stroke)	44	5.9	26	6.5	0.698
Chronic kidney disease stage 3 or above	34	4.5	29	7.3	0.057
Chronic obstructive airways disease	21	2.8	4	1.0	0.055
Depression	10	1.3	12	3.0	0.068
Diabetes	141	18.9	83	20.9	0.434
History of fall in the past 12 months	110	14.7	199	50.1	<0.001
Hepatitis B	15	2.0	7	1.8	1
Hyperlipidaemia	153	20.5	86	21.7	0.647
Hypertension	361	48.3	200	50.4	0.535
Hyperthyroidism	26	3.5	24	6.0	0.049
Ischemic heart diseases	61	8.2	17	4.3	0.013
Kyphosis	77	10.3	55	13.9	0.080
Malignancy within 5 years	40	5.3	24	6.0	0.685
Osteoarthritis knee	130	17.4	66	16.6	0.805
Peptic ulcer	56	7.5	19	4.8	0.080

^a^ *P* value was calculated by independent *t*-test

^b^ *P* value was calculated by chi-square test

The associations between each chronic disease per each SD decrease in handgrip strength in men and women are shown in Tables 2 and 3, respectively. In men, decreased handgrip strength was associated with increased odds of 12 chronic diseases. After adjusting for age, BMI, history of smoking, educational level and marital status, the associations between handgrip strength and stroke, CKD stage 3 or above, diabetes, history of fall in the past 12 months, and hyperthyroidism remained significant. After further adjusting for co-existing diseases, the associations with stroke (OR = 1.68, 95% CI = 1.10–2.58, *P* = 0.017), CKD stage 3 or above (OR = 2.76, 95% CI = 1.59–4.78, *P* < 0.001), and hyperthyroidism (OR = 1.92, 95% CI = 1.11–3.30, *P* = 0.019) remained significant, whereas the associations with anxiety (OR = 3.57, 95% CI = 1.46–8.77, *P* = 0.005) and chronic obstructive airways diseases (OR = 2.19, 95% CI = 1.05–4.55, *P* = 0.036) became significant.

**Table 2:** Binary logistic regression analysis of each SD reduction in handgrip strength with chronic diseases in men

Chronic diseases	Model 1		Model 2		Model 3	
	OR (95% CI)	*P* value	OR (95% CI)	*P* value	OR (95% CI)	*P* value
Anaemia	1.82 (1.09–3.05)	**0.022**	1.37 (0.74–2.55)	0.320	0.68 (0.28–1.63)	0.384
Anxiety	1.52 (0.84–2.75)	0.169	1.98 (0.98–4.00)	0.056	3.57 (1.46–8.77)	**0.005**
Cataract	1.51 (1.14–1.99)	**0.004**	1.30 (0.93–1.81)	0.127	1.21 (0.84–1.75)	0.312
Cerebral vascular accident (Stroke)	2.07 (1.49–2.87)	**<0.001**	1.85 (1.25–2.72)	**0.002**	1.68 (1.10–2.58)	**0.017**
CKD stage 3 or above	2.89 (1.97–4.24)	**<0.001**	2.71 (1.71–4.31)	**<0.001**	2.76 (1.59–4.78)	**<0.001**
COAD	2.48 (1.56–3.91)	**<0.001**	1.74 (0.98–3.09)	0.059	2.19 (1.05–4.55)	**0.036**
Depression	0.74 (0.37–1.48)	0.398	0.56 (0.24–1.32)	0.186	0.35 (0.11–1.10)	0.072
Diabetes	1.42 (1.17–1.73)	**0.001**	1.39 (1.09–1.76)	**0.007**	1.28 (0.99–1.66)	0.063
Hepatitis B	1.49 (0.87–2.53)	0.146	1.55 (0.82–2.93)	0.182	1.56 (0.78–3.12)	0.209
History of fall in the past 12 months	1.53 (1.23–1.90)	**<0.001**	1.44 (1.11–1.87)	**0.006**	1.31 (0.99–1.73)	0.059
Hyperlipidaemia	0.99 (0.81–1.19)	0.891	1.04 (0.83–1.31)	0.740	0.99 (0.76–1.28)	0.932
Hypertension	1.29 (1.10–1.51)	**0.001**	1.13 (0.93–1.37)	0.218	0.99 (0.80–1.22)	0.901
Hyperthyroidism	1.58 (1.05–2.38)	**0.029**	1.75 (1.08–2.82)	**0.024**	1.92 (1.11–3.30)	**0.019**
Ischemic heart diseases	1.49 (1.13–1.97)	**0.005**	1.30 (0.93–1.82)	0.120	1.32 (0.90–1.93)	0.157
Kyphosis	1.44 (1.12–1.85)	**0.004**	1.31 (0.97–1.76)	0.082	1.22 (0.88–1.67)	0.231
Malignancy within 5 years	1.47 (1.05–2.05)	**0.025**	1.17 (0.78–1.75)	0.460	1.16 (0.75–1.79)	0.499
Osteoarthritis knee	0.99 (0.81–1.21)	0.927	1.04 (0.82–1.32)	0.748	1.02 (0.78–1.32)	0.905
Peptic ulcer	1.08 (0.81–1.44)	0.608	1.06 (0.75–1.50)	0.743	0.90 (0.62–1.31)	0.570

*P* ≤ 0.05 values are in bold

Model 1: unadjusted model; model 2: adjusted for age, BMI, educational level, history of smoking and marital status; model 3: adjusted for age, BMI, history of smoking, educational level, marital status and other co-existing chronic diseases

*CKD* chronic kidney disease, *COAD* chronic obstructive airways disease

**Table 3:** Binary logistic regression analysis of each SD reduction in handgrip strength with chronic diseases in women

Chronic diseases	Model 1		Model 2		Model 3	
	OR (95% CI)	*P* value	OR (95% CI)	*P* value	OR (95% CI)	*P* value
Anaemia	1.82 (1.28–2.58)	**0.001**	1.65 (1.09–2.52)	**0.019**	1.83 (1.14–2.92)	**0.012**
Anxiety	1.09 (0.54–2.20)	0.802	1.16 (0.48–2.79)	0.742	1.46 (0.36–6.02)	0.597
Cataract	1.34 (1.12–1.60)	**0.001**	0.97 (0.78–1.22)	0.810	0.91 (0.71–1.17)	0.457
Cerebral vascular accident (stroke)	1.78 (1.29–2.46)	**0.001**	1.57 (1.06–2.32)	**0.023**	1.29 (0.78–2.11)	0.320
CKD stage 3 or above	1.68 (1.23–2.28)	**0.001**	1.03 (0.68–1.56)	0.899	1.04 (0.64–1.68)	0.868
COAD	2.09 (0.96–4.57)	0.063	1.99 (0.73–5.38)	0.176	4.93 (0.50–48.85)	0.172
Depression	1.00 (0.63–1.58)	0.992	0.82 (0.44–1.51)	0.525	0.74 (0.35–1.54)	0.420
Diabetes	1.46 (1.19–1.79)	**<0.001**	1.24 (0.97–1.58)	0.088	1.21 (0.90–1.63)	0.208
Hepatitis B	0.62 (0.34–1.14)	0.125	0.51 (0.22–1.14)	0.099	0.65 (0.26–1.64)	0.363
History of fall in the past 12 months	1.39 (1.17–1.64)	**<0.001**	1.43 (1.16–1.76)	**0.001**	1.44 (1.15–1.81)	**0.002**
Hyperlipidaemia	0.95 (0.79–1.15)	0.624	0.99 (0.78–1.25)	0.926	0.89 (0.68–1.17)	0.390
Hypertension	1.40 (1.19–1.66)	**<0.001**	1.07 (0.86–1.32)	0.543	1.02 (0.79–1.30)	0.906
Hyperthyroidism	0.95 (0.68–1.32)	0.762	1.06 (0.70–1.62)	0.771	1.11 (0.68–1.81)	0.689
Ischemic heart diseases	1.32 (0.90–1.94)	0.153	1.20 (0.76–1.90)	0.442	1.00 (0.57–1.78)	0.993
Kyphosis	1.83 (1.43–2.34)	**<0.001**	1.64 (1.23–2.19)	**0.001**	1.80 (1.32–2.46)	**<0.001**
Malignancy within 5 years	0.99 (0.71–1.38)	0.944	0.80 (0.52–1.23)	0.307	0.84 (0.52–1.36)	0.482
Osteoarthritis knee	1.00 (0.81–1.23)	0.987	0.98 (0.75–1.27)	0.878	1.05 (0.79–1.40)	0.733
Peptic ulcer	1.12 (0.78–1.62)	0.545	0.98 (0.63–1.54)	0.934	0.96 (0.57–1.62)	0.891

*P* ≤ 0.05 values are in bold

Model 1: unadjusted model; model 2: adjusted for age, BMI, educational level, history of smoking and marital status; model 3: adjusted for age, BMI, history of smoking, educational level, marital status and other co-existing chronic diseases

*CKD* chronic kidney disease, *COAD* chronic obstructive airways disease

In women, decreased handgrip strength was associated with increased odds of eight chronic diseases. After adjustment of age, BMI, history of smoking, educational level and marital status, the associations between handgrip strength and anaemia, stroke, history of fall in the past 12 months, and kyphosis remained significant. After further adjusted for co-existing diseases, low handgrip strength remained robustly associated with increased odds of anaemia (OR = 1.83, 95% CI = 1.14–2.92, *P* = 0.012), history of fall in the past 12 months (OR = 1.44, 95% CI = 1.15–1.81, *P* = 0.002), and kyphosis (OR = 1.80, 95% CI = 1.32–2.46, *P* < 0.001).

The results of multivariable analyses of chronological age and handgrip strength as markers of multimorbidity in men and women are shown in Table 4. In men, chronological age was associated with four chronic diseases, whereas handgrip strength was associated with five chronic diseases. In women, chronological age was associated with five chronic diseases, whereas handgrip strength was associated with three chronic diseases. Interestingly, in both men and women, the diseases associated with chronological age and handgrip strength were mutually exclusive.

**Table 4:** Odds ratio and 95% CI of chronic diseases per increase in age or per SD reduction in handgrip strength in the multivariable model

Chronic disease	Men (*n* = 748)				Women (*n* = 397)			
	Chronological age		HGS		Chronological age		HGS	
	OR (95% CI)	*P* value	OR (95% CI)	*P* value	OR (95% CI)	*P* value	OR (95% CI)	*P* value
Anaemia	0.97 (0.86–1.09)	0.605	0.68 (0.28–1.63)	0.384	0.99 (0.92–1.05)	0.652	1.83 (1.14–2.92)	**0.012**
Anxiety	0.96 (0.86–1.06)	0.424	3.57 (1.46–8.77)	**0.005**	0.81 (0.66–0.99)	**0.040**	1.46 (0.36–6.02)	0.597
Cataract	1.06 (1.01–1.11)	**0.025**	1.21 (0.84–1.75)	0.312	1.09 (1.05–1.13)	**<0.001**	0.91 (0.71–1.17)	0.457
Cerebral vascular accident (stroke)	1.03 (0.98–1.10)	0.272	1.68 (1.10–2.58)	**0.017**	1.02 (0.95–1.10)	0.586	1.29 (0.78–2.11)	0.320
CKD stage 3 or above	1.06 (0.99–1.14)	0.105	2.76 (1.59–4.78)	**<0.001**	1.14 (1.06–1.24)	**0.001**	1.04 (0.64–1.68)	0.868
COAD	0.92 (0.84–1.02)	0.100	2.19 (1.05–4.55)	**0.036**	0.95 (0.67–1.34)	0.752	4.93 (0.50–48.85)	0.172
Depression	1.12 (0.98–1.29)	0.102	0.35 (0.11–1.10)	0.072	0.96 (0.87–1.07)	0.509	0.74 (0.35–1.54)	0.420
Diabetes	1.00 (0.97–1.04)	0.864	1.28 (0.99–1.66)	0.063	1.02 (0.98–1.06)	0.361	1.21 (0.90–1.63)	0.208
Hepatitis B	1.00 (0.92–1.09)	0.921	1.56 (0.78–3.12)	0.209	0.94 (0.83–1.06)	0.295	0.65 (0.26–1.64)	0.363
History of fall in the past 12 months	1.03 (0.99–1.06)	0.180	1.31 (0.99–1.73)	0.059	0.97 (0.94–1.00)	0.064	1.44 (1.15–1.81)	**0.002**
Hyperlipidaemia	1.00 (0.97–1.04)	0.792	0.99 (0.76–1.28)	0.932	0.99 (0.96–1.03)	0.693	0.89 (0.68–1.17)	0.390
Hypertension	1.06 (1.03–1.09)	**<0.001**	0.99 (0.80–1.22)	0.901	1.06 (1.03–1.10)	**<0.001**	1.02 (0.79–1.30)	0.906
Hyperthyroidism	0.99 (0.92–1.05)	0.670	1.92 (1.11–3.30)	**0.019**	0.91 (0.85–0.97)	**0.004**	1.11 (0.68–1.81)	0.689
Ischemic heart diseases	1.07 (1.02–1.13)	**0.011**	1.32 (0.90–1.93)	0.157	1.09 (0.98–1.21)	0.135	1.00 (0.57–1.78)	0.993
Kyphosis	1.05 (1.01–1.10)	**0.025**	1.22 (0.88–1.67)	0.231	1.03 (0.98–1.07)	0.256	1.80 (1.32–2.46)	**<0.001**
Malignancy within 5 years	1.04 (0.98–1.10)	0.180	1.16 (0.75–1.79)	0.499	1.04 (0.98–1.11)	0.182	0.84 (0.52–1.36)	0.482
Osteoarthritis knee	1.01 (0.98–1.04)	0.598	1.02 (0.78–1.32)	0.905	1.00 (0.96–1.04)	0.841	1.05 (0.79–1.40)	0.733
Peptic ulcer	1.01 (0.96–1.06)	0.771	0.90 (0.62–1.31)	0.570	1.04 (0.96–1.12)	0.326	0.96 (0.57–1.62)	0.891

*P* ≤ 0.05 values are in bold

Model was mutually adjusted for age and handgrip strength T-score, and further adjusted for BMI, history of smoking, educational level, marital status and other co-existing chronic diseases

*HGS* handgrip strength

**Fig. 1:**
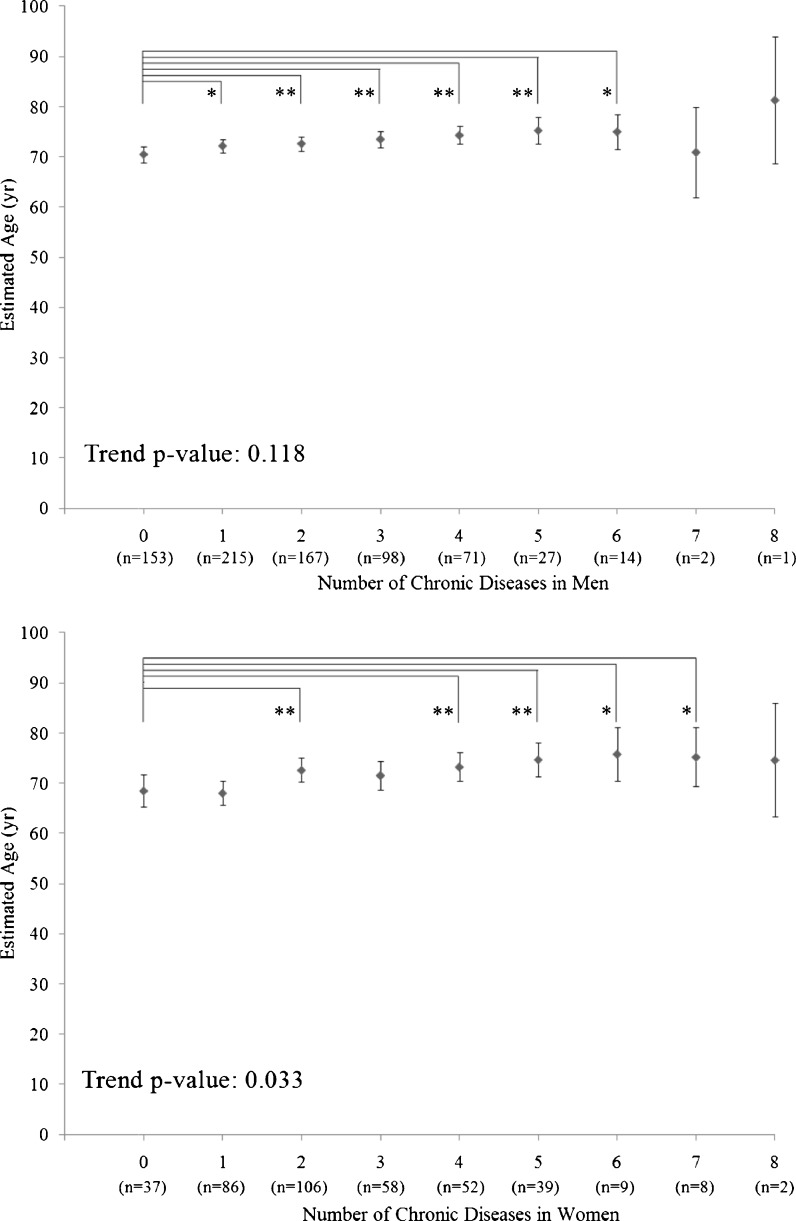
Mean age of subjects with various number of chronic diseases in men (*n* = 748; *upper panel*) and women (*n* = 397; *lower panel*). Values presented are means and 95% confidence intervals adjusted for handgrip strength, BMI, history of smoking, educational level and marital status. *P* values represent tests for linear trend in age across subjects with different number of chronic diseases. *0.01 < *P* ≤ 0.05; **P ≤ 0.01

**Fig. 2:**
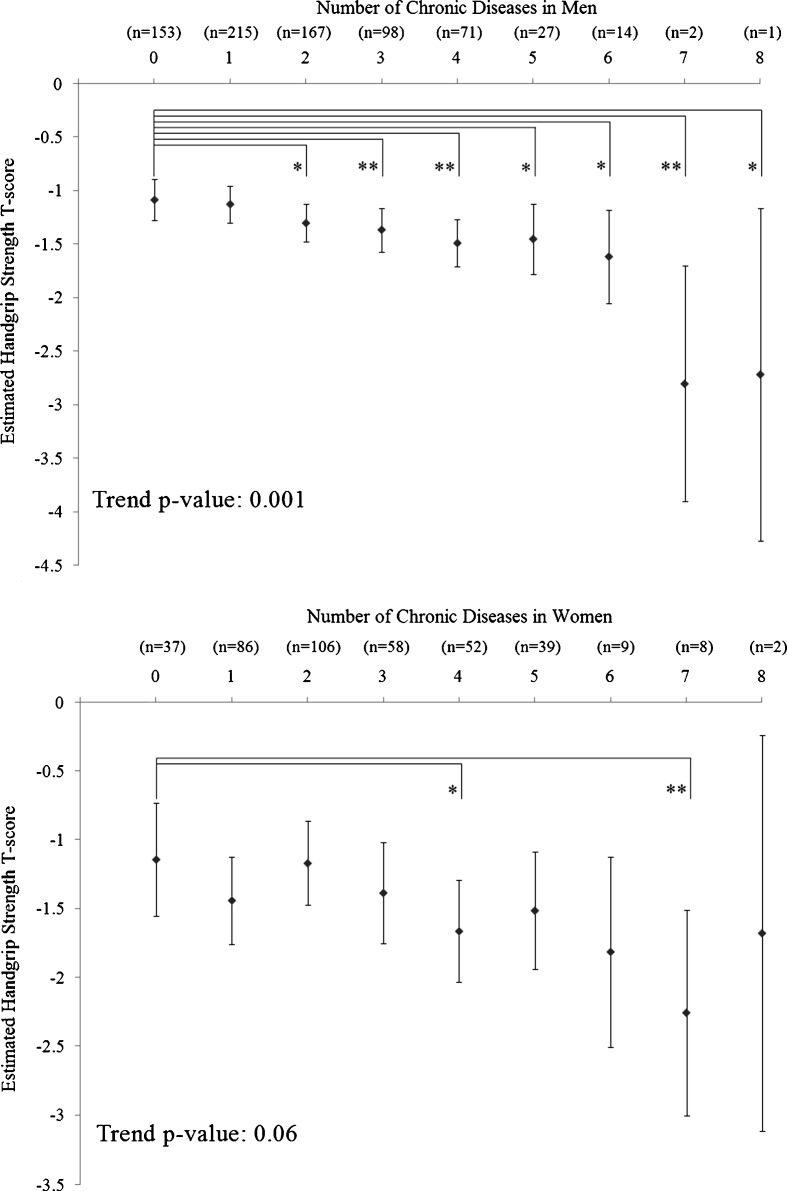
Mean handgrip strength T-score of subjects with various number of chronic diseases in men (*n* = 748; *upper panel*) and women (*n* = 397; *lower panel*). Values presented are means and 95% confidence intervals adjusted for age, BMI, history of smoking, educational level and marital status. *P* values represent tests for linear trend in age and handgrip strength *T*-score across subjects with different number of chronic diseases. *0.01 < *P* ≤ 0.05; **P ≤ 0.01

The results of multivariable ANCOVA analysis of age and handgrip strength as a marker of multimorbidity are shown in Figs. 1 and 2. For the analysis of age (Fig. 1), there was a significant positive linear trend between age and number of chronic diseases in women (trend, *P* = 0.033), but not in men (trend, *P* = 0.118; Fig. 1). In men, subjects without chronic disease were significantly younger than subjects having one to six chronic diseases. In women, subjects without chronic disease were significantly younger than subjects having two and four to seven chronic diseases. For the analysis of handgrip strength (Fig. 2), there was a significant negative linear trend between handgrip strength and number of chronic diseases in men (trend, *P* = 0.001), but not in women (trend, *P* = 0.06; Fig. 2). In men, subjects without chronic disease had significantly higher handgrip strength *T*-score than subjects having two to eight chronic diseases. In women, subjects without chronic disease had significantly higher handgrip strength than subjects having four, and seven chronic diseases.

## Discussion

To our best knowledge, this study is the first study demonstrating that handgrip strength is associated with multimorbidity, and that handgrip strength may be a more useful marker of multimorbidity than chronological age in men. Moreover, we also demonstrated that handgrip strength was associated with different chronic diseases, even when comorbidity and other confounding factors were taken into account. In previous studies, low handgrip strength has been linked with premature mortality in middle-aged and elderly subjects. Our current findings provide a possible explanation: premature mortality could be due to the presence of multiple chronic diseases.

It is a common perception that handgrip strength is related mainly to the muscular system (Filho et al. 2010). In this study, we showed that handgrip strength was associated with multiple chronic diseases after controlling for comorbidity. These diseases may be related but not necessarily restricted to muscular system, including anaemia, anxiety, CKD stage 3 or above, chronic obstructive airways disease, diabetes, hyperthyroidism, stroke, and kyphosis. It is not surprising that handgrip strength is significantly associated with a history of fall, as handgrip strength is an indicator of general muscle strength and associated with lower extremity strength (Norman et al. 2010; Samson et al. 2000; Xue et al. 2010). For other non-musculoskeletal diseases, it could be that handgrip strength may be associated with endocrine or other physiologic systems.

It has been reported that both subclinical inflammation and insulin resistance are risk factors for low muscle strength (Abbatecola et al. 2005; Barzilay et al. 2009; Schaap et al. 2009). In addition, a recent study suggested that long-term exposure to obesity is associated with poor handgrip strength later in life (Stenholm et al. 2011), further suggesting that low handgrip strength could be a marker reflecting the presence of subclinical inflammation and defects in glucose metabolism. It is well acknowledged that reduced muscle strength is a consequence of stroke. However, in a recent population-based cohort study of 1 million Swedish men, reduced handgrip strength was associated with increased risk of incident coronary heart disease and all strokes (Silventoinen et al. 2009), after adjustment of various confounding factors, such as birth cohort, age, BMI, height, blood pressure, and occupational socioeconomic position, suggesting that low handgrip strength is not only a marker, but also a predictor of coronary heart disease and stroke. Nevertheless, these observations suggested that handgrip strength is associated with multiple physiological systems and the mechanisms underlying handgrip strength deterioration, such as insulin resistance, hyperthyroidism (Harrison and Clausen 1998; Wang et al. 2006), increased interleukin-6 or reduced insulin-like growth factor I (IGF-I) (Barbieri et al. 2003), are common to other diseases.

In this study, the association patterns of handgrip strength with chronic diseases in men and women were very different. This could be due to the intrinsic differences between both genders, such as sex hormones. Earlier study suggested that sex hormone status is an important factor of handgrip strength in men but not in women (Baumgartner et al. 1999). Subsequently a number of studies have investigated the relationship between sex hormone and muscle mass or strength. Page et al. (2005) reported that exogenous testosterone improved handgrip strength in older men (Page et al. 2005). Conversely, in a randomized control trial study of Women’s Health Initiative, hormone therapy in postmenopausal women did not improve the grip strength (Michael et al. 2010). These observations are actually in agreement with an earlier randomized controlled trial which reported that sex steroid and growth hormone intervention significantly increased muscle strength in men, but not women (Blackman et al. 2002), suggesting that sex hormones may exert the effect on handgrip strength differently or through different mechanisms in men and women.

Handgrip strength appeared to be more important in determining multimorbidity than chronological age in men. Syddall et al. (2003) reported that grip strength was associated with more markers of frailty than chronological age in 717 men and women within a narrow age range (aged 64–74 years). Our study is in agreement with this study, as this observation was also observed in men and marginally in women. In men, we observed that chronological age was significantly associated with cataract, hypertension, ischemic heart diseases and kyphosis, whereas handgrip strength was significantly associated with anxiety, stroke, CKD stage 3 or above, chronic obstructive airways disease and hyperthyroidism. The importance of handgrip strength was further strengthened by the ANCOVA analysis, showing that there was a statistically significant inverse linear trend association between handgrip strength and number of co-existing chronic diseases. However, the linear trend was not observed between chronological age and number of co-existing chronic diseases, suggesting handgrip strength could be a marker of multimorbidity and independent of the effect of age and other confounding factors.

Chronological age, however, appeared to be more important in determining multimorbidity than handgrip strength in women. Although it is well established that the presence of chronic diseases increases with age, older age is an un-modifiable risk factor. Handgrip strength, however, is an objectively measured parameter that could potentially reflect multimorbidity. Research has shown that low handgrip strength was linked to premature mortality (Sasaki et al. 2007); therefore, improving handgrip strength or muscle strength could be a possible way to reduce the likelihood of premature mortality by reducing the number of chronic diseases present. Although the current study did not establish the causality between handgrip strength and overall health, previous studies suggested that enhancing handgrip strength may be beneficial to physiological systems other than muscular system. For example, isometric handgrip training for 5 to 6 weeks has been demonstrated to reduce resting arterial blood pressure (Peters et al. 2006; Taylor et al. 2003), which is probably mediated by the autonomic control (Taylor et al. 2003) and myocardial mechanisms (Millar et al. 2009). In addition, a number of cytokines, growth hormones and hormones are produced by muscle, such as myostatin and IGF-I. Myostatin is mainly produced by skeletal muscle, and it inhibits myoblast differentiation and proliferation (McPherron et al. 1997). Subjects with chronic heart failure had higher expression of myostatin in skeletal muscle (Lenk et al. 2011) and higher serum level of myostatin latent complex (George et al. 2010), after 12 weeks of exercise training, myostatin in skeletal muscle of chronic heart failure patient was significantly reduced (Lenk et al. 2011). Although circulating IGF-I is mainly produced by the liver and partly produced by skeletal muscle, previous study showed that muscle IGF-I has a more significant role in growth and development than hepatic IGF-I (Klover and Hennighausen 2007; Yakar et al. 1999). Improving muscle strength by means of resistance training may be a feasible strategy for improving general health and decrease risk for multiple chronic diseases.

There are several strengths in the current study. Our study was confined to people from southern China, therefore minimizing the heterogeneity of results due to the large differences among people with different lifestyles and/or genetic components. We also adjusted for BMI in our analysis in order to overcome the issue related to the influence of body weight on handgrip strength (Foley et al. 1999). Since occurrences of some diseases are highly correlated, we adjusted for other co-existing diseases in model 3 (Tables 2 and 3) in order to take account for it.

Several limitations of this study warrant discussion. First, the cross-sectional design does not establish a casual relation between handgrip strength and multimorbidity examined in this study. Although the self-reported disease status was validated using the computerized patient record system of the Hong Kong Hospital Authority, possibility of false negative still exists for those asymptomatic and undiagnosed chronic diseases. Moreover, we cannot determine whether the observed handgrip strength was pathological or they were the consequences of having multimorbidity. Nevertheless, several prospective studies suggested that handgrip strength may precede some chronic diseases. In addition, multimorbidity and frailty are closely related, since handgrip strength is considered as one of the criteria of frailty in Study of Osteoporotic Fractures frailty index (Fried et al. 2001), we did not evaluate how frailty might affect our current findings. Although we have adjusted several confounding factors such as BMI, educational level and marital level (Marengoni et al. 2008; Taylor et al. 2010) in the analysis, we were unable to adjust for socioeconomic status and waist circumferences in the analysis, since these two factors were not included in our study.

In our questionnaire, we set all unknown or unsure answers to missing, thus leading to a high prevalence of missing data in our current study. Yet we found no material differences between the groups of subjects who were included and those excluded from the current study on several key variables such as age, handgrip strength *T*-score, and BMI. Hence, we believe the reduced number of subjects included in the study resulted in lowered statistical power. Finally, since we tested the associations between handgrip strength and 18 chronic diseases, an alternative p value of 0.0028 (0.05/18 tests) may be considered as the significant level to account for multiple testing. Thus, results from Tables 2, 3 and 4 should be interpreted with caution.

In conclusion, handgrip strength is associated with multiple chronic diseases in men and women, even accounted for age, BMI, history of smoking, educational level, marital status and comorbidity. It is also associated with multimorbidity. In addition, handgrip strength appeared to be a more useful marker of multimorbidity than chronological age in men. Since it is an objective parameter assessing muscle strength, which is affecting and controlled by multiple physiological system, future clinical trial is warranted to investigate its usefulness in intervention or prevention of multimorbidity, and hence possibly preventing premature mortality.

## Electronic supplementary material

Below is the link to the electronic supplementary material.

Fig. S1 Study recruitment and eligibility flow chart (DOC 63 kb)

Table S1 Categories of diseases studied based on World Health Organization’s International Classification of Diseases (10th Revision, version for 2007, http://apps.who.int/classifications/apps/icd/icd10online/) (DOC 56 kb)
