# Supplementary figures and images for: Association of handgrip strength with chronic diseases and multimorbidity: A cross-sectional study
Source: Age (Dordr). 2012 Feb 8;35(3):929–41. doi: 10.1007/s11357-012-9385-y (PMC3636411; doi:10.1007/s11357-012-9385-y)

Supplementary Figure 1. Study recruitment and eligibility flow chart.


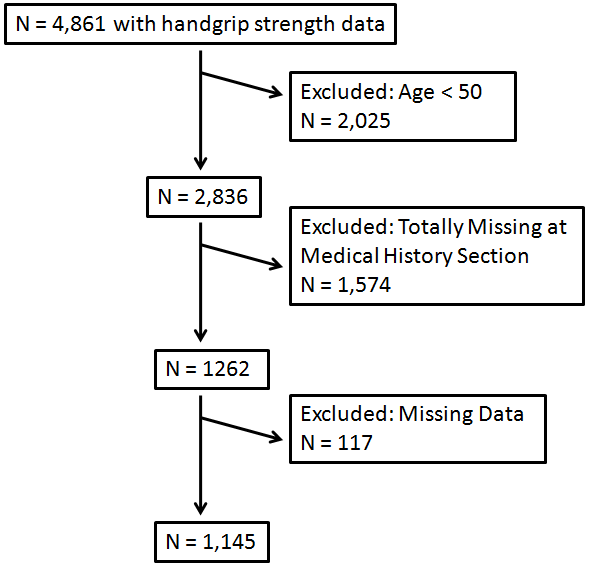

Supplement: Supplementary file 1 — Study recruitment and eligibility flow chart (DOC 63 kb) [file 11357_2012_9385_MOESM1_ESM.doc]
