# Supplementary material for: Association of handgrip strength with chronic diseases and multimorbidity: A cross-sectional study
Source: Age (Dordr). 2012 Feb 8;35(3):929–41. doi: 10.1007/s11357-012-9385-y (PMC3636411; doi:10.1007/s11357-012-9385-y)
Supplement: Supplementary file 2 — Categories of diseases studied based on World Health Organization’s International Classification of Diseases (10th Revision, version for 2007, http://apps.who.int/classifications/apps/icd/icd10online/) (DOC 56 kb) [file 11357_2012_9385_MOESM2_ESM.doc]

Supplementary Table 1. Categories of diseases studied based on World Health Organization’s International Classification of Diseases (10^th^ Revision, version for 2007, http://apps.who.int/classifications/apps/icd/icd10online/)

| **Chronic diseases** | **Categories based on World Health Organization’s International Classification of Diseases** | **ICD-10 Diagnostic Code(s)** |
| --- | --- | --- |
| Anaemia | diseases of the blood and blood-forming organs and certain disorders involving the immune mechanism | D50-D64 |
| Anxiety | mental and behavioural disorders | F41 |
| Cataract | diseases of the eye and adnexa | H25-H26 |
| Cerebral vascular accident (Stroke) | diseases of the circulatory system | I64 |
| CKD stage 3 or above | diseases of the genitourinary system | N03 |
| Chronic obstructive airways disease | diseases of the respiratory system | J44 |
| Depression | mental and behavioural disorders | F32-F33 |
| Diabetes | endocrine, nutritional and metabolic diseases | E10-E14 |
| History of fall in the past 12 months | symptoms, signs and abnormal clinical and laboratory findings, not elsewhere classified | R29.6 |
| Hepatitis B | certain infectious and parasitic diseases | B18 |
| Hyperlipidaemia | endocrine, nutritional and metabolic diseases | E78 |
| Hypertension | diseases of the circulatory system | I10 |
| Hyperthyroidism | endocrine, nutritional and metabolic diseases | E05 |
| Ischemic heart diseases | diseases of the circulatory system | I20-I25 |
| Kyphosis | diseases of the musculoskeletal system and connective tissue | M40 |
| Malignancy within 5 years | neoplasms | C00-D48 |
| Knee osteoarthritis | diseases of the musculoskeletal system and connective tissue | M17 |
| Peptic ulcer | diseases of digestive system | K27 |
